# Genomic Characterization and Phylogenetic Position of Two New Species in *Rhabdoviridae* Infecting the Parasitic Copepod, Salmon Louse (*Lepeophtheirus salmonis*)

**DOI:** 10.1371/journal.pone.0112517

**Published:** 2014-11-17

**Authors:** Arnfinn Lodden Økland, Are Nylund, Aina-Cathrine Øvergård, Steffen Blindheim, Kuninori Watanabe, Sindre Grotmol, Carl-Erik Arnesen, Heidrun Plarre

**Affiliations:** 1 Department of Biology, University of Bergen, 5020 Bergen, Norway; 2 SLRC-Sea Lice Research Center, Institute of Marine Research, 5817 Bergen, Norway; 3 SLRC-Sea Lice Research Center, Department of Biology, University of Bergen, 5020 Bergen, Norway; 4 Firda Sjøfarmer AS, 5966 Eivindvik, Norway; Columbia University, United States of America

## Abstract

Several new viruses have emerged during farming of salmonids in the North Atlantic causing large losses to the industry. Still the blood feeding copepod parasite, *Lepeophtheirus salmonis*, remains the major challenge for the industry. Histological examinations of this parasite have revealed the presence of several virus-like particles including some with morphologies similar to rhabdoviruses. This study is the first description of the genome and target tissues of two new species of rhabdoviruses associated with pathology in the salmon louse. Salmon lice were collected at different Atlantic salmon (*Salmo salar*) farming sites on the west coast of Norway and prepared for histology, transmission electron microscopy and Illumina sequencing of the complete RNA extracted from these lice. The nearly complete genomes, around 11 600 nucleotides encoding the five typical rhabdovirus genes N, P, M, G and L, of two new species were obtained. The genome sequences, the putative protein sequences, and predicted transcription strategies for the two viruses are presented. Phylogenetic analyses of the putative N and L proteins indicated closest similarity to the Sigmavirus/Dimarhabdoviruses cluster, however, the genomes of both new viruses are significantly diverged with no close affinity to any of the existing rhabdovirus genera. *In situ* hybridization, targeting the N protein genes, showed that the viruses were present in the same glandular tissues as the observed rhabdovirus-like particles. Both viruses were present in all developmental stages of the salmon louse, and associated with necrosis of glandular tissues in adult lice. As the two viruses were present in eggs and free-living planktonic stages of the salmon louse vertical, transmission of the viruses are suggested. The tissues of the lice host, Atlantic salmon, with the exception of skin at the attachment site for the salmon louse chalimi stages, were negative for these two viruses.

## Introduction

The salmon louse, *Lepeophtheirus salmonis*, feeding on mucus, skin and blood of the host, is a serious problem during farming of the Atlantic salmon, *Salmo salar*, in Norway [1], [2]. The life cycle of the salmon louse includes an egg/embryonic stage, two free-living stages, one free-living parasitic stage, and five parasitic stages on the surface of the salmonid host. The salmon louse is attached to the host via a frontal filament during the first two parasitic stages (chalimi stages), while moving freely on the surface of the host during the two preadult and the adult stage [3]. The reproduction of *L. salmonis* in salmon farms and its subsequent release of larvae into the surrounding sea are also recognized as a problem for wild salmonids, *S. salar* and *S. trutta*, along the Norwegian coast [4]. Several control strategies are being used including neurotoxins, hydrogen peroxide, and the use of cleanerfish. The latter method has a limited effect and represents an additional danger of introducing other fish pathogens (ex. *Paramoeba perurans*) into the salmon cages [5]. The development in the industry is moving towards a critical situation, where the requirements (from the Norwegian Food Authorities, NFA) of a low number of lice on each farmed salmon has led to an increased use of neurotoxins, resulting in the emergence of multiple resistance against these chemicals in the lice populations [6]. Unless new groups of anti-parasitica are developed in the coming years, the aquaculture industry could be facing a critical situation where they are not able to meet the requirements from the NFA and environmental organizations that to a certain degree represent the public opinion on salmon farming.

This development, combined with new advances in biotechnology, may lead to a future use of lice pathogens in the control of this salmonid ectoparasite. One possibility is the use of lice viruses, or their constitutive parts, into novel lice control agents or strategies. There are no published studies of viruses in *L. salmonis*, but several studies have focused on viruses in other crustaceans with a main focus on viruses in commercially important decapods [7], [8], [9], [10], [11], [12], [13], [14], [15], [16], [17], [18], [19], [20], [21], [22], [23], [24], [25], [26], [27], [28], [29]. These studies have shown the presence of members of several different virus families among the crustaceans, including both DNA and RNA viruses.

Studies using transmission electron microscopy on tissues from *L. salmonis* collected from farmed Atlantic salmon in western Norway have shown the presence of different morphs of virus-like particles (A. Nylund, pers. obs.). These viruses, based on the virion morphology and site of assembly, include both DNA and RNA viruses, and the associated histopathology suggests that they may have a significant negative effect on the salmon louse. These viruses, or some of them, could possibly be developed as a tool for future lice control in salmonid aquaculture, but before that can be a reality there are some major problems that have to be resolved. Prior experiences with insect viruses have shown that improvements in the virus efficacy, large scale production and perceived safety will be needed if the lice viruses are to play a major role in the control of this parasite. Knowledge about the genome of these viruses is needed to develop specific and sensitive methods for detection and identification. Fast and safe methods for detection and identification are a necessity for the work towards developing lice viruses as a strategy for control of *L. salmonis*. This study describes the genome of two new species of rhabdoviruses present in salmon louse, the target tissues and the possible virion morphology.

## Materials and methods

### Material

Lice (*Lepeophtheirus salmonis*) showing signs of internal changes were collected at five different farming sites on the west coast of Norway in the summer-autumn periods in 2008 – 2013, and transported live to the Fish Disease Research Laboratory at the University of Bergen. A selection of the individuals were sampled both for histology/transmission electron microscopy (TEM) and RNA/DNA extraction, while a large bulk of lice, all the different developmental stages and egg strings, were collected for RNA extraction only. Small subsamples of lice tissues, showing signs of morphological changes, were stored at −80°C for later culture of possible viruses present.

Tissues (gills, skin, heart and kidney) from Atlantic salmon (*Salmo salar*) infected with different stages of *L. salmonis* were collected from a farm in western Norway. The skin tissues were taken from the surface areas where chalimi stages of the lice were attached and from skin areas on the head and behind the dorsal fins, i.e. areas with frequent presence of preadult and adult lice stages. These tissues and different developmental stages of the salmon louse were used for RNA extraction and real time RT PCR.

### Histology and TEM

Tissues from lice or one half of the lice cut along the longitudinal axis were fixed in a modified Karnovsky fixative. The fixed tissues were used for histological studies and transmission electron microscopy (TEM). The tissues were processed and sectioned as described in Steigen et al. [30].

### RNA extraction

**Figure 1:**
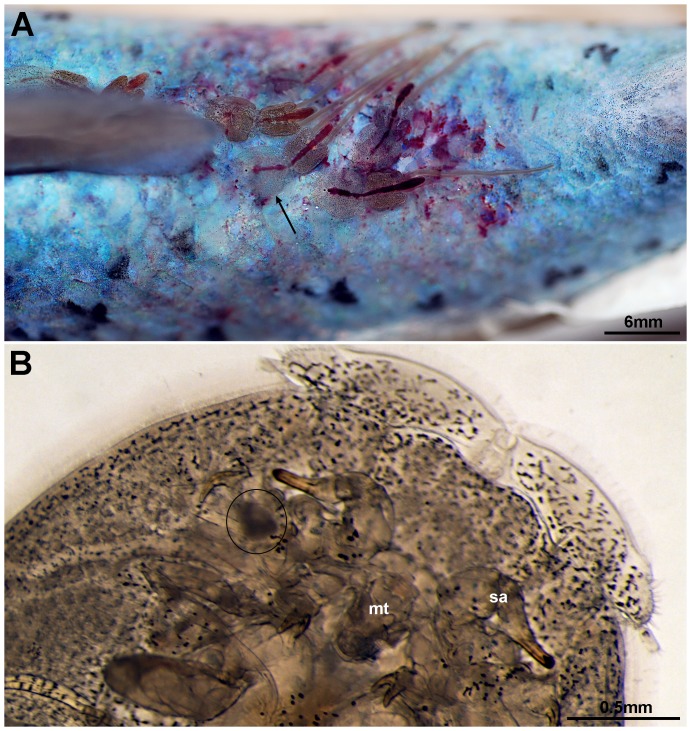
Adult female salmon lice, *Lepeophtheirus salmonis* (arrow), feeding on Atlantic salmon (A). An area of reduced transparency (ring) in the cephalothorax in the vicinity of the second antenna (sa) adult lice (B). Mouth tubule (mt).

Salmon lice (*L. salmonis*), showing areas of reduced transparency in the cephalothorax in the vicinity of the second antenna (Figure 1), were collected for RNA extraction. The occluded areas, the area from behind the mouth tubule to the anterior of the lice, including the tissues with low light transparency, were used for the RNA extraction. RNA was extracted from individual samples as described by Steigen et al. [30].

The RNA was used for Illumina sequencing, RT PCR and real time RT PCR. The latter method was used for the detection of two rhabdovirus genomes detected in salmon louse after Illumina sequencing.

RNA was also extracted from the collected Atlantic salmon tissues and from the different developmental stages of the salmon louse. The RNA was used for real time RT PCR, PCR and Sanger sequencing.

### Illumina sequencing

Total RNA was isolated from the anterior part of the cephalothorax, including the mouth tubule, from five salmon lice collected from five different farms in western Norway. The RNA was pooled and sent to BaseClear (BaseClear Group, Netherlands) for Illumina (Illumina Casava pipeline version 1.8.3) sequencing. At BaseClear a library was created using Illumina TruSeq RNA library preparation kit (Illumina). No polyA capture was used. cDNA synthesis was then performed on fragmented dsRNA, and DNA adapters were ligated to both ends of the DNA fragments before being subjected to PCR-amplification. Prior to sequencing the library was checked on a Bioanalyzer (Agilent) and quantified. The library was sequenced on a full Illumina HiSeq 2500 genome analyzer using a paired-end protocol. The resultant reads were quality checked and low quality reads were removed using the Illumina Chastity filtering. An in-house filtering protocol was used to remove reads containing adapters and/or PhiX control signal. The reads were assembled using the “De novo assembly” option of the CLC Genomics Workbench version 7.0 (CLCbio). This resulted in 10 463 sequences with an average sequence size of 544 bp and a total sum of 5 698 290 bp. Selected sequences were translated using ExPASy's online translation tool (http://web.expasy.org/translate/) and the BLASTP algorithm of the BLAST suite was used to identify the sequences.

Two sequences were identified as possible members of *Rhabdoviridae*. These two sequences, No9 (Accession no: KJ958535) and No127 (Accession no: KJ958536), were used as template for production of primers used to confirm these virus sequences through Sanger sequencing.

### Real time RT PCR

Two real time RT PCR assays (Taqman probes) were developed based on the putative nucleoprotein gene sequences of No9 and No127 (Table 1). The assays were optimized for relative quantification. An assay targeting the elongation factor from salmon louse was used as internal control [31]. During real time RT PCR on salmon tissues an assay targeting the elongation factor alpha from Atlantic salmon was used as internal control [32].

**Table 1:** Primers and probes for Taqman real time RT PCR assays targeting the N protein gene of the two salmon louse rhabdoviruses, LSRV-No9 and LSRV-No127.

Code	Sequence	Position
No9-NF	5′-TCC AAC AGA TCT CCT TAC TCA GTC A -3′	922–946
No9-Nprobe	5′- CGC CAA TCC CTT ATT -3′	948–962
No9-NR	3′- TCC AAT GAT ATG GAC CCA CAT G – 5′	987–966
No127-NF	5- CTA TGG AGC CAT CGG AGG TTA T -3′	873–894
No127-Nprobe	5′- ACC TGG GTG ACT CTT -3′	896–910
No127-NR	5′- CAA GAT CTC AGT CGA GAC GGA AT -3′	934–912

The position of the primer and probes are related to the ORF of the N protein gene of the two viruses.

### Determination of 5′ end terminal sequences of the N protein genes of the two Rhabdovirus from *L. salmonis*

The RNA used in the RNA ligase-mediated amplification of 5′ cDNA ends (GeneRacer Kit version L, Invitrogen) of the two lice rhabdoviruses, No9 and No127, N protein genes were obtained from the anterior part of lice with glandular pathology. The protocol given by the manufacturer was followed using the primers (GeneRacer 5′primer) included in the kit and virus genome specific primers for 5′end race (No9-5′endGSP; CGT TGT TGG GAC CTT CAC GGA CAC A, and No127-5èndGSP; GGC TGG TGT TAC GAG TAT TGA TTT). The final PCR products were cloned into pCR4-TOPO vector (Invitrogen) and sequenced. Sequences were assembled and analysed using the VectorNTI 9.0 software.

### Culture system for lice viruses

The only known culture system for these two viruses is the host itself, *L. salmonis*. There are no established cell cultures available from salmon louse or other caligids. Since a range of rhabdoviruses can be cultured in BF2 cells it was decided to test four different cell cultures from fish to see if any of these were susceptible for the two identified rhabdoviruses. In theory, it is possible that these viruses could use the salmon host as a vector for transmission between individual lice, which means that there was a slight possibility that existing cell cultures from salmonids could be susceptible.

The following cell cultures were tested as possible culture systems for these two putative rhabdoviruses; BF-2 (ATCC CCL91), ASK cells [33], CHSE-214 [34], and RT-Gill-W1 cells [35]. The cells were cultured in 25 cm^2^ tissue culture flasks at 20°C in Eagle's minimum essential medium (EMEM) supplemented with foetal bovine serum (10%), L-glutamine (4 mM), Non-Essential amino acids and gentamicin (50 µg ml−1). The cells were sub-cultured weekly and formed monolayers within 4–7 days.

For virus propagation, cell culture medium was removed from cell monolayers, and sterile-filtered homogenates from positive salmon lice, diluted 1∶10 in serum depleted medium (2% FBS, 4 mM L-glutamine, non-essential amino acids, gentamicin), was added. The cells were incubated at 15°C for 4–5 weeks, or until cytopathic effect (CPE) was observed. The supernatant from the first passage was passed to new cultures, and the cell layers from the first and second inoculation were tested for presence of the two viruses by real time RT PCR.

### *In situ* hybridization

*In situ* hybridization was performed according to Dalvin et al. [36], with some modifications as described in Tröße et al. [37]. The digoxigenin labelled (DIG-labelled) sense and antisense RNA probes were made with primers listed in Table 2.

**Table 2:** The digoxigenin labelled (DIG-labelled) sense and antisense RNA probes were made with primers listed.

Name	Sequence
RhabNt F1(LSRV-No127Npro)	GGAGCCATCGGAGGTTATGACC
RhabNt R1(LSRV-No127Npro)	AAGGGGCCGTGTCAATCCTA
RhabNs F1(LSRV-No9-Norf)	TTCTCCCGAACCGACATGGA
RhabNs R1(LSRV-No9-Norf)	AGGGGATTGGCGGTGACTGA

### Phylogeny

The sequence data were preliminarily identified by GenBank searches done with BLAST (2.0) and the Vector NTI Suite software package was used to obtain multiple alignments of sequences. To perform pairwise comparisons of the two rhabdovirus sequences from the salmon louse, the multiple sequence alignment editor GeneDoc (available at: www.psc.edu/biomed/genedoc) was used for manual adjustment of the sequence alignments. Selected sequences from other members of the family *Rhabdoviridae*, already available on the EMBL nucleotide database, were included in the alignments. Members of the genera *Cytorhabdovirus*, *Novirhabdovirus* and *Nucleorhabdovirus* were excluded because of their large amino acid difference from the two louse viruses. Ambiguously aligned regions were removed using Gblocks [38]. This resulted in sequence alignments of 256 and 1630 amino acids for the N and L proteins, respectively. Phylogenetic relationships were determined using the maximum-likelihood (ML) method available in TREE_PUZZLE 5.2 (available at: http://www.tree-puzzle.de), employing the VT [39] model of amino acid substitution. Quartet puzzling was used to choose from the possible tree topologies and to simultaneously infer support values for internal branches. Quartet trees are based on approximate maximum likelihood values using the selected model of substitution and rate heterogeneity. The robustness of each node was determined using 20 000 puzzling steps. Phylogenetic trees were drawn using TreeView [40].

### Protein analysis

The Compute pI/Mw tool in ExPASy was used to calculate the theoretical pI (isoelectric point) and Mw (molecular mass) of the putative proteins coded by the ORFs in the genome of the two rhabdoviruses present in *L. salmonis*. The Phobius server were used to predict N-terminal signal peptide, ectodomain, transmembrane region, and C-terminal cytoplasmic tail in the topology analyses of the glycoprotein genes of the two rhabdoviruses. The ExPASy Bioinformatics Resource Portal (http://www.expasy.org/proteomics) was used for identification of putative glycosylation and phosphorylation sites. Motifscan (http://myhits.isb-sib.ch/cgi-bin/motif_scan) were used on the L protein from the two viruses to predict catalytic domains.

## Results

### Virus morphology

**Figure 2:**
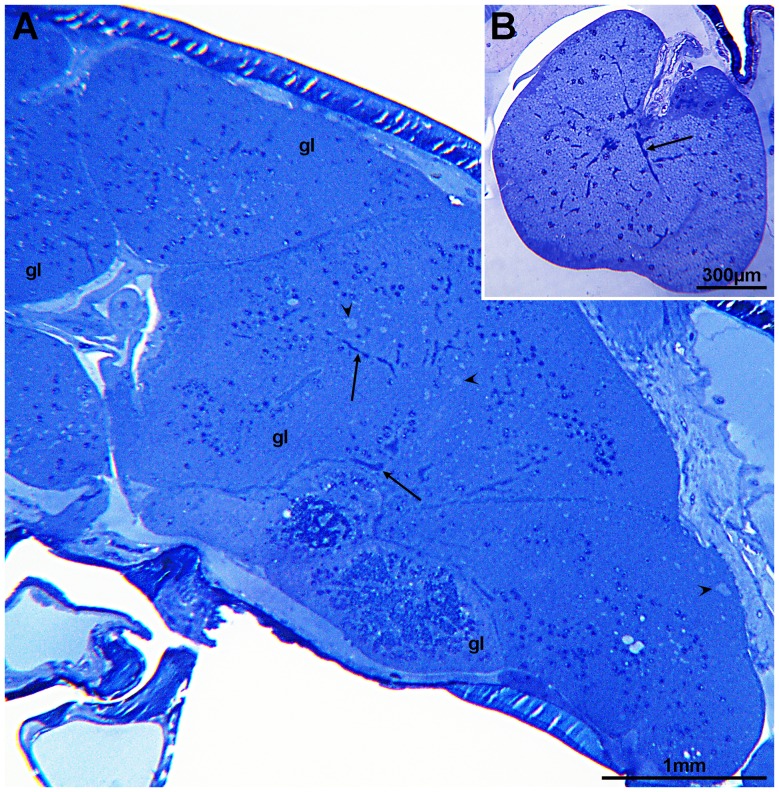
Sections of virus infected glands (gl) situated between the second antenna and the mouth tubule. Accumulation of virions (arrows) and viroplasm-like structures (arrow heads) (A). Virus-infected gland opening in the mouth tubule of the lice (B). Accumulation of virions (arrow).

**Figure 3:**
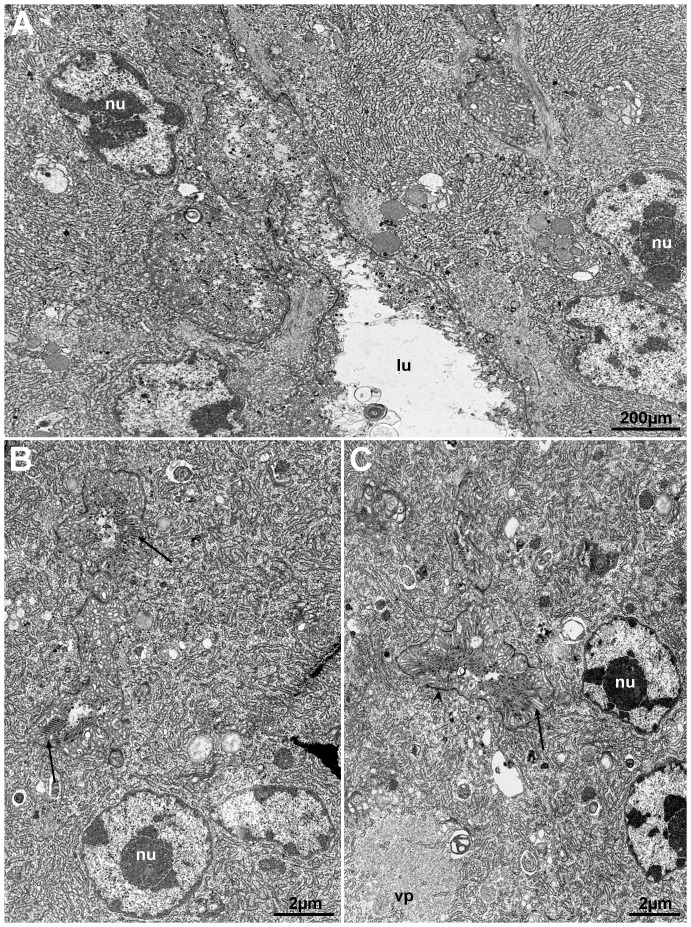
Multinucleated (nu) gland cells with channels containing virus-like particles (arrows) and amorphic material (A and B). C) This figure shows viroplasm (vp) in the vicinity of a channel containing virus-like particles (arrow). Note the accumulation of electron dense material (arrow head) on the inside of the cell membrane. Nucleus (nu).

**Figure 4:**
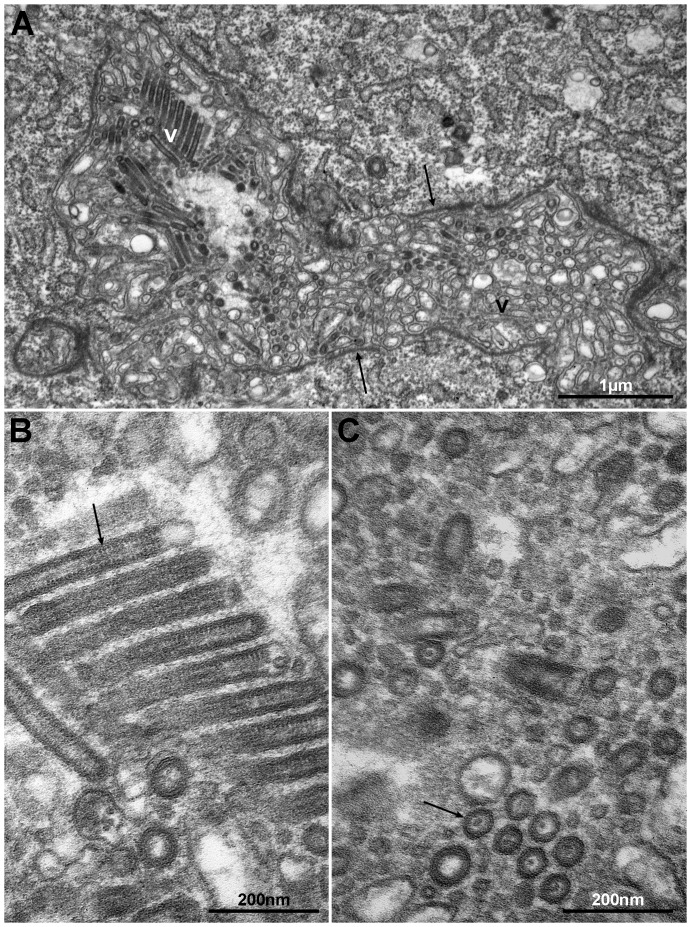
Section through a channel (lu) in a gland cell containing large amounts of virus-like particles (V). Note the accumulation of electron dense material (arrows) on the inside of the cell membrane (A). The virus particles (arrow) are enveloped, rod-shaped or bacilliform, and appear as being cross-striated in tangential longitudinal sections (B). Transverse section through virus particles showing surrounding unit membrane and an electron dense core (C).

Salmon lice (*L. salmonis*), showing areas of reduced transparency in the cephalothorax in the vicinity of the second antenna (anterior part of the cephalothorax), were collected from farmed Atlantic salmon (Figure 1). Sectioning of these occluded areas showed that they consisted of glandular tissues (Figure 2). In some lice the tissues were necrotic or completely disintegrated. One set of glands seems to open in the vicinity of the mouth tubule of the lice. Transmission electron microscopy (TEM) of the glandular tissues showed that they are most likely syncytia or tissue consisting of large multinucleated cells. Large amounts of virus-like particles were seen budding from cellular membranes, surface membranes or membranes of the Golgi/endoplasmatic reticulum system (Figure 3). Modified areas, possibly viroplasm, were observed in the cytoplasm of the glandular cells (Figure 3C). The virus particles were enveloped and rod-shaped or bacilliform with a diameter of 55 nm and a maximum length of 425 nm (Figure 4). The nucleocapsid seemed to exhibit a helical symmetry since in longitudinal tangential sections of the virions they appear as being cross-striated (spacing about 8.5–9.0 nm) (Figure 4B).

### Genome

Illumina sequencing of the RNA from lice with glandular pathology and presence of virus-like particles, generated two nearly complete rhabdovirus genomes, *Lepeophtheirus salmonis* rhabdovirus No9 (LSRV-No9) and *L. salmonis* rhabdovirus No127 (LSRV-No127), with lengths of 11 681 and 11 519 nucleotides, respectively. These two sequences were used as template for construction of primers that were used for RT PCR and Sanger sequencing of the two virus genomes. No errors in the two genomes generated by Illumina sequencing were detected. Both viruses (Accession numbers: KJ958535, KJ958536) contain five open reading frames in the negative sense genome in the order ‘3-N-P-M-G-L-5’ also found in other rhabdoviruses.

### The 3-leader and 5-trailer regions

**Figure 5:**
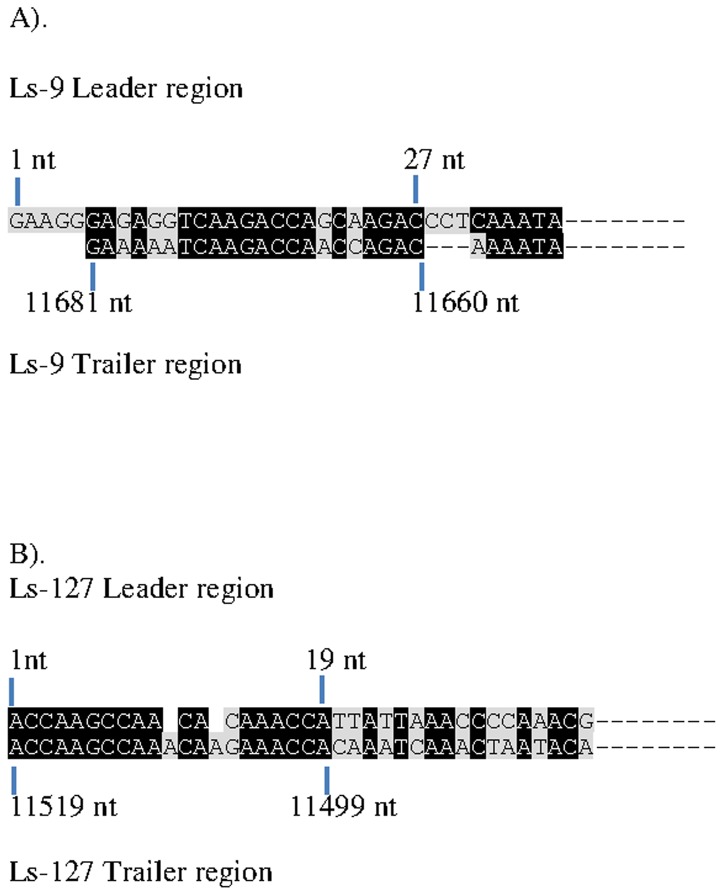
The non-translated 3′-end and 5′-end regions of the two viruses may not be complete but still exhibit inverse complementarity. The first nucleotides of the leader of LSRV-No9 aligned with the inverse complementary last nucleotides of the trailer (A). The first nucleotides of the leader of LSRV-No127 aligned with the inverse complementary last nucleotides of the trailer (B).

The Illumina sequencing generated a leader region of LSRV-No9 and LSRV-No127 consisting of the first 61 and 70 nucleotides, respectively, with trailer regions composed of 122 and 58 nucleotides, respectively. The non-translated 3′-end and 5′-end regions of the two viruses may not be complete but still exhibit inverse complementarity. The first 27 nt of the leader of LSRV-No9 show 63.0% identity to the last 22 nucleotides of the trailer, while the first 19 nucleotides in the leader of LSRV-No127 show 89.5% identity to the last nucleotides in the trailer (Figure 5). The identities of the leader and trailer region from LSRV-No9 compared to the same regions in LSRV-No127 are 42.6% (61 nt compared) and 47.6% (63 nt compared), respectively. The leader, first 24 nucleotides, of LSRV-No9 and LSRV-No127 show 50.0% and 91.7% identity to Vesicular stomatitis New Jersey virus (NJ89GAS, Accession no: JX121110), respectively.

### Gene junctions

The distances between translation stop and start codons in the gene junctions of the two viruses range from 47 (G-L) to 136 (N-P) nucleotides and from 41 (G-L) to 271 (N-P) nucleotides in the genomes of LSRV-No-9 and LSRV-No127, respectively. The untranscribed intergenic regions, the gene junctions between the polyadenylation sequence and the transcription initiation sequence, of the two lice rhabdoviruses vary in length (0 to 6 nt). The nucleotide sequences of the intergenic regions are not conserved between the two viruses and are also different from that of other related rhabdovirus genera (Table 3).

**Table 3:** Leader and trailer regions for isolates Ls9 and Ls127.

Isolate	Gene	Leader	Trailer	Intergenic sequence	
Ls9	N	AACAG	TATGAAAAAAA		
Ls9	P	AACAA	TATGAAAAAAA	N-P	CAGT
Ls9	M	AACAA	TATGAAAAAAA	P - M	-
Ls9	G	AACAA	TATGAAAAAAA	M - L	CGGTTT
Ls9	L	AACAG	TATGAAAAAAA	G - L	TCT
Ls127	N	TAAGAA	TATGAAAAAAA	-	
Ls127	P	TAAGAA	TATGAAAAAAA	N - P	CT
Ls127	M	TAAGAA	TATGAAAAAAA	P - M	CCTC
Ls127	G	TAAGAA	TAAGAAAAAAA	M - G	CTAT
Ls127	L	CAAGAA	TATGAAAAAAA	G - L	T

Conserved transcription initiation (TI) and transcription termination/polyadenylation (TTP) sequences flank each gene to direct transcription of capped and polyadenylated mRNAs.

The putative transcription termination and polyadenylation signal, based on its homology to other rhabdoviruses, is conserved in the genomes of the two salmon louse viruses and comprises the motif TATG(A)^7^ with the exception of the transcription stop/polyadenylation signal of the G protein gene of LSRV-No127 which is TAAG(A)^7^ (Table 3).

The potential start sequence in the genome of LSRV-No9 is not conserved and the same sequence, AACAA, can only be found in the start of the P, M and G protein genes (Table 3). The start sequence of the N and L protein genes is AACAG. The start of the N protein gene was determined by 5-end RACE. The junction between the P/M genes in LSRV-No9 differs from the other junctions in these two viruses in that the transcriptional start signal of the M gene seems to start with the last two nucleotides of the transcriptional stop signal of the P gene, or, as an alternative, it precedes the transcriptional stop signal of the P gene resulting in a possible overlap of 27 nt.

The transcription initiation site sequences, expected to occur shortly after the previous transcription termination signal, seem to be TAAGAA in the genome of LSRV-No127 with the exception of the transcription initiation of the L protein gene, which seems to be CAAGAA (Table 3). The start of the N protein gene was determined by 5-end RACE.

### Protein genes

To annotate the coding sequences it has been assumed that each open reading frame (ORF) starts at the first AUG occurring after the previous transcription termination sequence, and that it continues to the first stop codon. The G protein gene is in reading frame one, the N, P and L protein genes are in reading frame two and the M protein gene is in reading frame three in the genome of LSRV-No9, while the N, P, M and G genes are in reading frame three and the L gene in reading frame one in the genome of LSRV-No127.

#### N gene

The 5′ ends of the N protein gene of the two salmon louse viruses were obtained using the GeneRacer Kit (Invitrogen) for full-length RNA ligase-mediated amplification of 5′ cDNA ends. The N gene in LSRV-No9 is 1691 nt long from the putative transcriptional start signal (AACAG) to the transcription termination signal (TATG(A)^7^), and contains a single ORF of 1491 nt encoding a putative protein of 497 amino acids (Table 4). The calculated molecular mass (Mw) of the protein is 56.8 kDa with an isoelectric point (pI) of 5.8. The N gene of LSRV-No9 also contains a 199-nt 3′-UTR of unknown function between the stop codon and the polyadenylation signal. Amino acid sequence comparisons with other rhabdoviruses using BLAST search show that LSRV-No9 shares the highest identity (28.0–33.0%) and similarity with members of viruses belonging to the Dimarhabdovirus and Sigma virus groups. However, the N protein of LSRV-No9 shows 89.9% nucleotide identity (97.2% amino acid similarity) to a possible rhabdovirus nucleoprotein (Accession no: BT077702) obtained from salmon lice (*L. salmonis*) in the Pacific Ocean (Canada).

**Table 4:** Predicted genes and putative proteins of LSRV-No9 and LSRV-No127.

Protein	Gene length (nt)	ORF (nt)	5′-UTR (nt)	3′-UTR (nt)	Protein (aa)
**No9**					
**N**	1691	1491	93	106	497
**P**	994	888	28	78	296
**M**	763	675	18	72	225
**G**	1659	1596	28	35	532
**L**	6380	6351	12	17	2117
**No127**					
**N**	1680	1398	69	213	466
**P**	926	789	57	80	263
**M**	736	657	23	56	219
**G**	1657	1626	20	11	542
**L**	6376	6288	30	58	2096

The N gene of LSRV-No127 is 1680 nt long from the putative transcription initiation site (TAAGAA) to the polyadenylation signal (TATG(A)^7^) containing a single ORF consisting of 1398 nt encoding a putative protein of 466 aa (Table 4). The calculated Mw of the protein is 52.8 kDa with a pI of 5.9. The identity of the nucleotide and putative amino acid sequences of the N protein of LSRV-No9 compared to LSRV-No127 are 48.7% and 25.6%, respectively.

The N proteins of LSRV-No9 and LSRV-No127 contain 26 and 31 potential phosphorylation sites, and the sequences, _306_GISNRSPYSV_315_ and _288_GISAKSPYSV_297_, respectively. These sequences are relatively conserved with the RNA binding motif (G(L/I)SXKSPYSS) present in vesiculoviruses, ephemeroviruses and lyssaviruses.

#### P gene

The putative LSRV-No9 P gene is 994 nt long and contains a single ORF of 888 nt encoding a putative protein of 296 aa, while the LSRV-No127 P gene is 926 nt long with a single ORF of 789 nt encoding a putative protein of 263 amino acids (Table 4). The calculated Mw of these two proteins are 32.6 kDa and 30.3 kDa with pI of 5.0 and 5.3, respectively. The P proteins of LSRV-No9 and LSRV-No127 contain 19 and 15 potential phosphorylation sites, respectively. Based on the predicted phosphorylation pattern it appears that both LSRV-No9 and LSRV-No127 P proteins contain a non-phosphorylated stretch in the centre, from amino acids 49–161 and 95–142, respectively. The two putative P protein sequences share no clear homology with the P proteins from other rhabdoviruses, while the amino acid similarity between the two viruses is 33.4%.

#### M gene

The M gene in LSRV-No9 is 763 nt long and contains a single ORF of 675 nt encoding a putative protein of 225 aa with calculated Mw of 25.1 kDa and a pI of 7.8. Amino acid sequence comparison with other rhabdoviruses, BLAST search, shows that it shares 25% identity with *Scophthalmus maximus* rhabdovirus, SMRV (ADU05404), and no significant match with other rhabdoviruses.

The M gene in LSRV-No127 is 736 nt long and with a single ORF of 657 nt encoding a putative protein of 219 amino acids with a calculated Mw of 24.0 kDa and a pI of 8.7. The identity and similarity of the putative amino acid sequences of LSRV-No9 compared to LSRV-No127 are 27.6% and 46.2%, respectively, while a BLAST search using the LSRV-No127 putative M protein gives identities in the range 21–23% with the M protein from Flanders virus (AGV98721), *Anguillid rhabdovirus* (AFJ94645), *Perch rhabdovirus* (YP007641365).

Both the predicted M proteins from LSRV-No9 and LSRV-No127 contain several phosphorylation sites, 14 and 18, respectively.

#### G gene

The G gene in LSRV-No9 is 1659 nt long and contains a single ORF of 1596 nt encoding a putative protein of 532 amino acids with a calculated Mw of 59.7 kDa and a pI of 6.7 (Table 4). Topology analyses using the Phobius server predict a transmembrane region spanning from amino acid 478–503 and a C-terminal cytoplasmic tail from aa 501–532. The protein is predicted to contain four putative N-glycosylation sites, _33_NGTT, _249_NQSC, _350_NSTL, and _445_NASI, respectively. Amino acid sequence comparisons with other rhabdoviruses, BLAST search, show that this virus ORF shares the highest identity (22.0–23.0%) and similarity with that of Spring viraemia of carp virus (Genus *Sprivivirus*).

The G gene of LSRV-No27 is 1657 nt long containing a single ORF consisting of 1626 nt encoding a putative protein of 542 aa with a calculated Mw of 62.2 kDa and a pI of 7.3. Topology analyses using the Phobius server predict an N-terminal signal peptide (aa 1–24, N-region aa 1–3, H-region aa 4–15, C-region aa 16–24), an ectodomain from aa 25–486, a transmembrane region spanning from amino acid 487–511, and a C-terminal cytoplasmic tail from aa 512–542. The protein is predicted to contain two putative N-glycosylation sites, _16_NLSI and _410_NSSD, respectively. The identity of the nucleotide sequence and the similarity of the putative amino acid sequences of LSRV-No9 compared to LSRV-No127 are 31.3% and 46.4%, respectively. BLAST searches show that LSRV-No127 shares the highest identity (24.0%) with a virus isolated from tick or bat, Kolente virus (Accession no: AHB08864, unclassified Rhabdovirus) which possibly belongs to the Dimarhabdovirus group. However, the G protein of LSRV-No127 shows 50.9% nucleotide identity (39.8% amino acid similarity) to a possible rhabdovirus glycoprotein (Accession no: BTO75815) obtained from *Caligus rogercresseyi* in the Pacific Ocean (Chile).

#### L gene

The last gene in the genome of the two salmon louse rhabdoviruses, the L protein gene, shows a clear affinity to other members of *Rhabdoviridae*, with the closest affinity (>35.0% identity) to the Dimarhabdoviruses and members of the genus *Sigmavirus*. The full length L proteins from LSRV-No9 and LSRV-No127 are closest to each other (40.4% identity) and to the L protein from turbot rhabdovirus, SMRV (>38.9%), and VSV (>38,8%) (Table 5). The *Sigmavirus* (>35.9%) and BEFV (>35.4%) are slightly more distant.

**Table 5:** Percent amino acid identity of Ls9 and Ls127 L protein domains and subdomains compared with a selection of related rhabdoviruses.

Virus	Entire	Blocks % identity						Subdomains block III (%)			
	L	I	II	III	IV	V	IV	III-A	III-B	III-C	III-D
**Ls9**											
VSV	38.8	**40.3**	64.4	**56.3**	46.2	53.2	56.5	**92.3**	88.5	**90.0**	**30.8**
SVCV	37.8	33.5	60.6	55.8	45.6	53.7	62.4	**92.3**	92.3	80.0	**30.8**
PFRV	37.6	35.1	59.6	55.8	45.0	53.7	60.0	**92.3**	92.3	**90.0**	**30.8**
PRV	38.0	36.1	59.6	52.7	46.8	56.7	58.3	**92.3**	92.3	**80.0**	**30.8**
SMRV	**38.9**	35.6	**65.4**	53.6	**52.0**	**61.0**	58.8	**92.3**	88.5	**90.0**	**30.8**
BEFV	36.1	35.1	61.5	50.9	39.2	52.8	50.6	84.6	88.5	80.0	23.1
TIBV	34.7	31.4	58.6	53.1	42.7	51.9	**67.9**	69.2	80.8	80.0	30.8
DURV	36.0	38.2	**65.4**	52.2	46.8	46.3	63.1	76.9	92.3	80.0	30.8
WONV	36.0	38.2	59.6	51.3	48.5	54.1	52.4	**92.3**	**100.0**	80.0	15.4
NGAV	36.5	31.9	62.5	53.6	44.4	55.0	57.1	84.6	88.5	**90.0**	15.4
MOUV	28.2	27.2	52.9	44.6	38.6	38.1	41.7	69.2	69.2	80.0	23.1
KOLEV	35.2	36.1	62.5								
SIGMAV	35.3	34.6	56.7	47.8	41.5	52.8	49.4	76.9	80.8	80.0	**30.8**
**Ls127**											
VSV	39.0	40.3	**63.5**	**53.6**	**55.0**	48.9	**60.0**	**100.0**	84.6	**90.0**	30.8
SVCV	38.1	36.6	58.7	51.8	48.5	51.1	54.1	**100.0**	84.6	80.0	30.8
PFRV	37.3	37.2	59.6	52.2	49.7	49.4	54.1	**100.0**	84.6	90.0	30.8
PRV	37.7	37.7	61.5	50.9	52.0	54.5	47.6	84.6	84.6	**80.0**	30.8
SMRV	**39.1**	35.6	62.4	47.3	54.4	**55.0**	56.5	**100.0**	84.6	**90.0**	30.8
BEFV	35.4	34.0	59.6	50.4	44.4	47.2	44.7	**100.0**	84.6	80.0	30.8
TIBV	34.1	33.0	55.8	47.3	47.4	46.3	58.3	61.5	76.9	80.0	23.1
DURV	35.8	**40.8**	**63.5**	48.7	51.5	46.3	59.5	76.9	84.6	80.0	23.1
WONV	35.1	35.1	62.5	48.2	53.2	51.5	48.8	84.6	92.3	80.0	15.4
NGAV	36.8	35.6	58.7	52.2	52.0	51.9	50.0	84.6	84.6	90.0	15.4
MOUV	28.4	28.3	50.0	41.5	43.3	39.0	40.5	61.5	65.4	80.0	23.1
KOLEV	36.1	37.2	58.7								
SIGMAV	35.9	37.7	55.8	46.9	46.8	48.9	50.6	76.9	76.9	80.0	**38.5**
Ls9	**49.4**	**53.4**	**68.3**	**67.9**	**66.7**	**62.8**	**69.4**	**76.9**	**92.3**	**100**	**69.2**

*Vesiculovirus* (VSV  =  Vesicular stomatitis virus (ABP01784)), *Sprivivirus* (SVCV  =  Spring viraemia of carp virus (ABW24037) and PFRV  =  Pike fry rhabdovirus (ACP28002)), *Perhabdovirus* (PRV  =  Perch rhabdovirus (AFX72892)), *Ephemerovirus* (BEFV  =  Bovine ephemeral fever virus (NP065409)), *Sigmavirus* (Sigma  =  Sigma virus (AFV52407)), *Tibrovirus* (TIBV  =  Tibrogargan virus (YP007641376)), *Tupavirus* (DURV  =  *Durham virus* (ADB88761)). Unassigned (SMRV  =  Turbot rhabdovirus (ADU05406), WONV  =  Wongabel virus (YP002333280), NGAV  =  Ngaingan virus (YP003518294), MOUV  =  Moussa virus (ACZ81407), KOLEV  =  Kolente virus (AHB08865)).

The L gene from LSRV-No9 is 6380 nt long and contains a single ORF of 6351 nt encoding a putative protein of 2117 aa, while the L gene from LSRV-No127 is 6376 nt long with a single ORF of 6288 nt encoding a putative protein of 2096 amino acids (Table 4). The calculated Mw of these two proteins are 241.8 kDa and 240.7 kDa with pI of 8.5 and 8.7, respectively.

The L gene is the most conserved in the family *Rhabdoviridae* and is structured into six conserved blocks that contain motifs for the structure and function of the L protein [41]. Pairwise alignments of the LSRV-No9 and LSRV-No127 L proteins with L proteins of selected members of Dimarhabdovirus and *Sigmavirus* show a pattern that conforms to the given conserved blocks. Block II is the most conserved of the major domains and block I is the least conserved showing identities at the same level as seen for the entire L protein (Table 5).

The first conserved amino acid motif, DYxLNSP, in the L proteins of the rhabdoviruses compared is found in position 46–52 and 39–45 of LSRV-No9 and LSRV-No127, respectively. Three amino acid motifs, LMxKD (LSRV-No9 residue 237–241, LSRV-No127 residues 231–235), SFRHxGHP (LSRV-No9 res. 359–366, LSRV-No127 res. 353–360), and LASDLA (LSRV-No9 res. 395–400, LSRV-No127 res. 389–394), are highly conserved among the rhabdoviruses included in the alignment of block I.

Block II is highly conserved among the rhabdoviruses compared and the KERELK motif present in *Vesiculovirus* is found as _535_KEREVK_540_ and _529_KEREMK_534_ in LSRV-No9 and LSRV-No127, respectively. This motif has been shown to be involved in the positioning and binding of RNA template and the KEREMK motif is also present in other rhabdoviruses like Tibrogargan virus, Wongabel virus, and Flanders virus. LSRV-No9 share this motif, _535_KEREVK_540_, with Ngaingan virus.

Within block III the subdomain III-A is the most conserved, while subdomain III-D shows lower amino acid identity than the overall identity for the complete L protein. The GGLEGLR motif and the sequence LAQGDNQVI (with the invariant peptide QGDNQ), the latter in position 715–723 in LSRV-No9 and 709–717 in LSRV-No127, could correspond to motifs B and C, in block III which is important for the polymerase function. Using motifscan (http://myhits.isb-sib.ch/cgi-bin/motif_scan) on the L protein from LSRV-No9 and LSRV-No127 a predicted catalytic domain between amino acids 603–791 and 587–785, respectively, is detected.

The conserved domains, the RNA polymerase domain, mRNA capping-region (block V), and a methyltransferase region, are also present in both the salmon louse viruses. The conserved motif GSxT-(60–70 aa)-HR in block V, which is essential for mRNA capping activity could correspond to the sequences _1162_GSKT-69aa-HR_1236_ and _1153_GSKT-69aa-HR_1227_ in the L protein of LSRV-No9 and LSRV-No127, respectively. The conserved motif, IKRA (present in *Vesiculovirus*) was also present in both the louse viruses as LKRA (position LSRV-No9: 1181–1184, LSRV-No127: 1175–1178).

Block VI showed the GxGxG motif as GDGSG in both LSRV-No9 (res. 1673–1677) and LSRV-No127 (res. 1666–1670) which could play a role of polyadenylation or protein kinase activity.

### Phylogeny

To reveal the relationships of the two louse viruses, LSRV-No9 and LSRV-No127, to other members of the family Rhabdoviridae, phylogenetic trees based on the L and N proteins were generated. Members of the genera *Cytorhabdovirus*, *Novirhabdovirus*, and *Nucleorhabdovirus* were excluded due to their large divergence which reduced the phylogenetic resolution, and the lyssaviruses were also removed from the alignment of the N protein due to high divergence. The ambiguously aligned regions in the alignments were removed using Gblocks resulting in sequence alignments of the L and N protein of 1630 and 256 amino acids, respectively.

**Figure 6:**
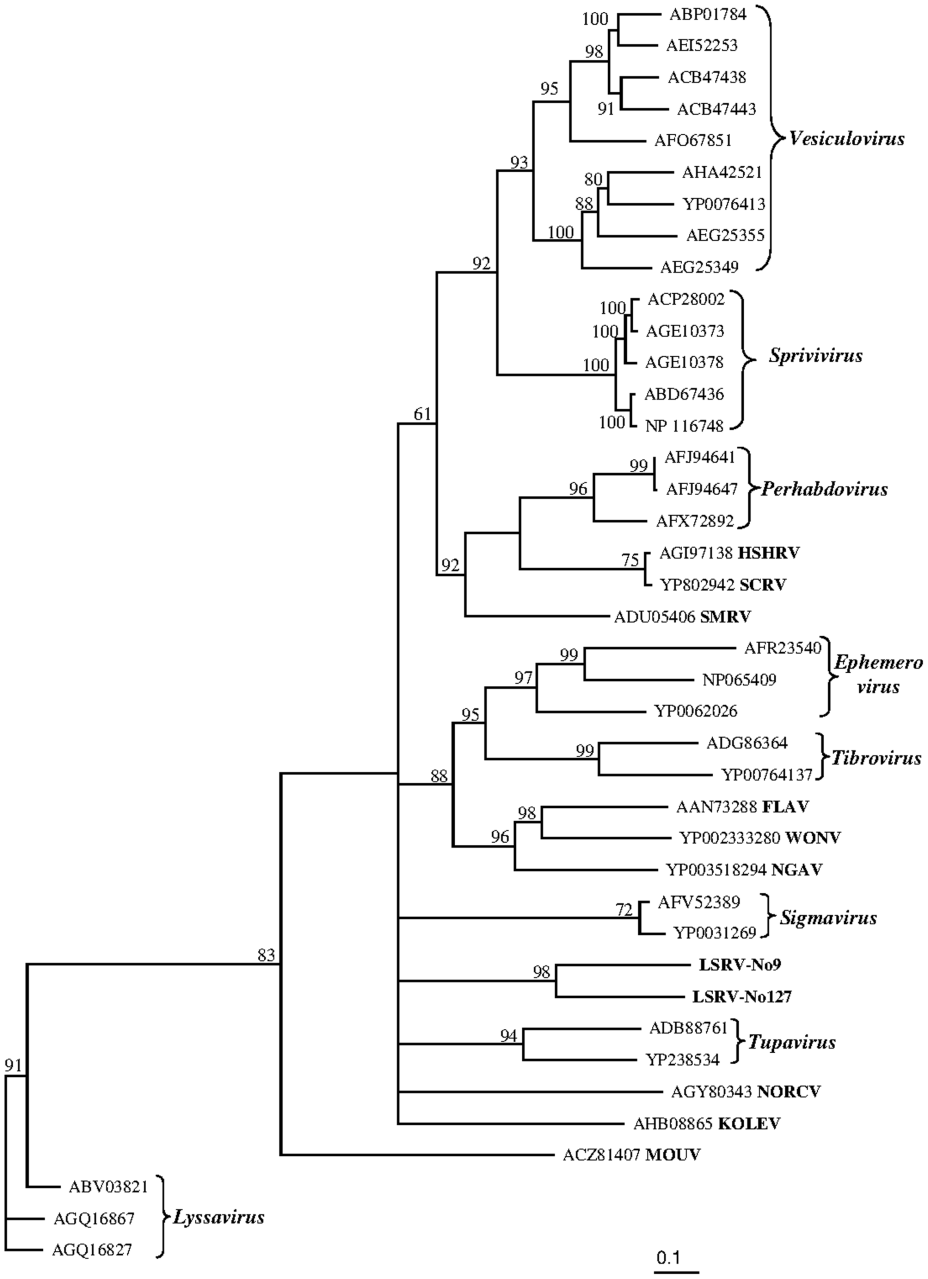
Phylogenetic position of two *Rhabdoviridae*, LSRV-No9 (Accession no: KJ958535) and LSRV-No127 (Accession no: KJ958536), obtained from salmon louse (*L. salmonis*) in relation to other rhabdoviruses based on analysis of the L protein sequences after removal of ambiguously aligned regions using Gblocks [38]. The evolutionary relationship is presented as maximum likelihood trees based on 1630 aa from the complete alignment of the L protein amino acid sequences. Branch lengths represent relative phylogenetic distances according to maximum likelihood estimates based on the VT matrix [39]. The scale bar shows the number of amino acid substitutions as a proportion of the branch lengths.

**Figure 7:**
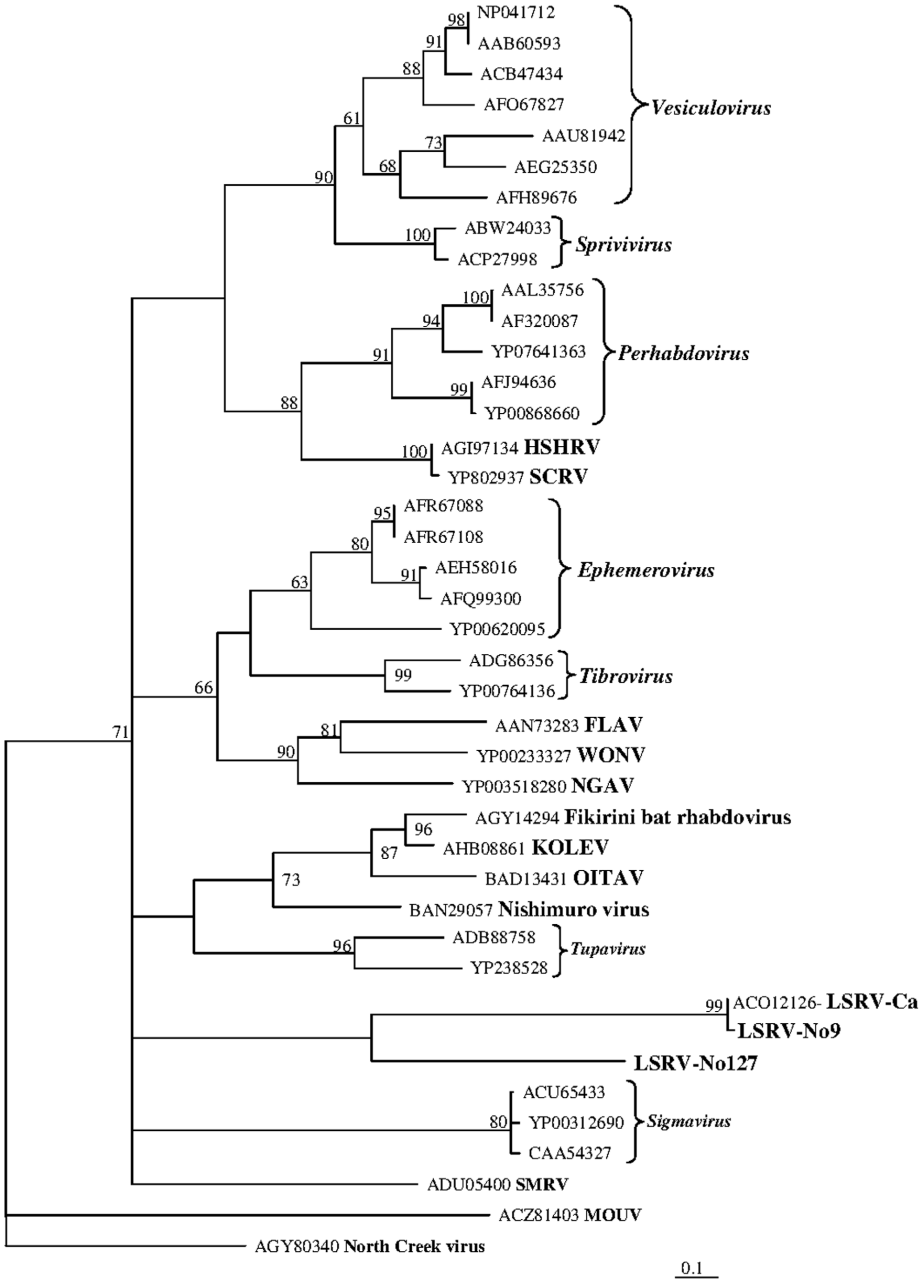
Phylogenetic position of two *Rhabdoviridae*, LSRV-No9 (Accession no: KJ958535) and LSRV-No127 (Accession no: KJ958536), obtained from salmon louse (*L. salmonis*) in relation to other rhabdoviruses based on analysis of the N protein sequences after removal of ambiguously aligned regions using Gblocks [38]. The evolutionary relationship is presented as maximum likelihood trees based on 256 aa from the complete alignment of the N protein amino acid sequences. Branch lengths represent relative phylogenetic distances according to maximum likelihood estimates based on the VT matrix [39]. The scale bar shows the number of amino acid substitutions as a proportion of the branch lengths.

In the phylogeny based on the L protein the two viruses from salmon louse, LSRV-No9 and LSRV-No127, group in a distinct clade with uncertain affinity to the other rhabdovirus genera included in the study and distant from the lyssaviruses (Figure 6). The phylogeny based on the N protein shows even less affinity between the two salmon louse viruses and no clear affinity to any of the assigned genera included in the study (Figure 7). However, LSRV-No9 groups closely with a rhabdovirus N protein sequence (Accession no: ACO12126) obtained from salmon louse (*L. salmonis*) in the Pacific Ocean (Canada).

### Screening

Selected tissues from Atlantic salmon (N = 70) infected with *L. salmonis* and different developmental stages of the salmon louse (N = 165), including egg strings, were tested for presence of both rhabdoviruses, LSRV-No9 and LSRV-No127, using real time RT PCR.

All life stages of the salmon louse tested positive for both rhabdoviruses, but the largest amounts of virus RNA were detected in adult lice (Ct values as low as 12 were obtained for both viruses). Virus RNA were also present in the eggs and embryos. All tissues (skin, gills, heart, kidney) from the Atlantic salmon were negative or only slightly positive (CT values >30) with the exception of skin tissues surrounding the attachment site for the chalimi stages. The Ct values at the attachment site were in the range between 22 and 30 indicating presence of substantial amounts of virus RNA.

### *In situ* hybridization

**Figure 8:**
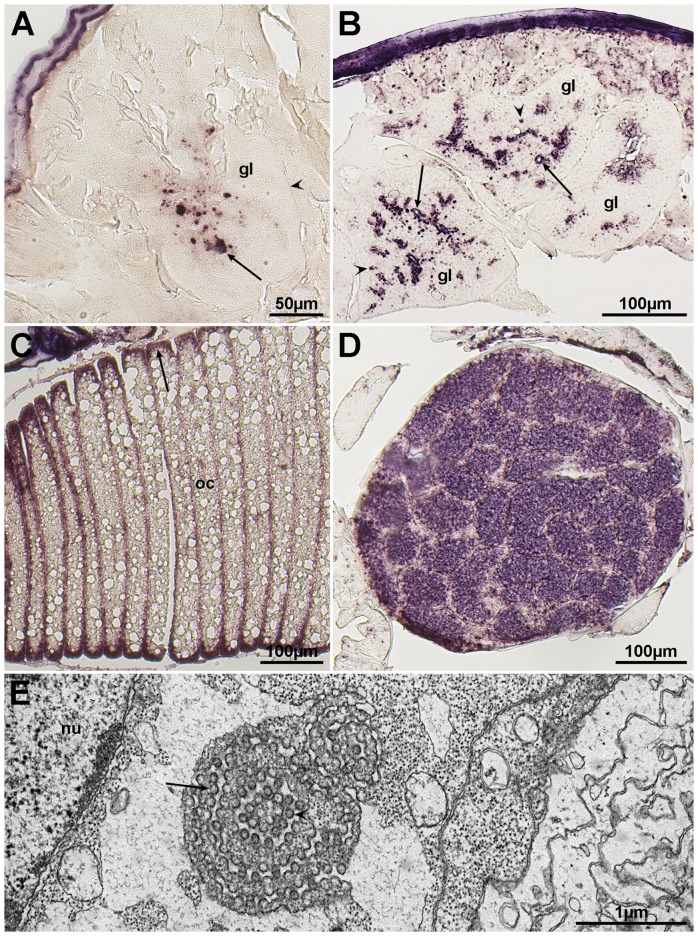
In situ hybridization for localization of LSRV genomes and mRNAs encoding the putative N protein. *In situ* hybridization with an antisense probe targeting mRNA encoding the N protein of LSRV-No9 results in patches of staining (arrow) within an exocrine gland (gl), where the arrowhead is pointed at the gland capsule. These patches may represent viroplasm (A). A sense probe targeted at the LSRV-No127 genome induces coloring in or around gland (gl) secretory ducts, which are indicated by arrows. This may reflect viral budding through the cytoplasmic membrane and the presence of mature virions within the lumen of the duct, as shown by TEM (Figure 3 and 4). Patches of staining (arrowheads) in the cytoplasm may reflect viroplasm (B). Utilization of an antisense probe aimed at LSRV-No127 mRNA encoding the N protein, results in staining (arrow) of cytoplasm at the periphery of oocytes (oc), and within the ovary (figures C and D). TEM picture of putative virions budding (arrow) into the lumen of ER (E). It is not known if these spherical virus-like particles (arrow head) are connected to any of the two rhabdoviruses. Nucleus of the ovary cell (nu).

The two viruses had similar tissue tropism (Figure 8). Staining was observed in glands, subcuticular tissue and, in some instances in peripheral cytoplasm of skeletal muscle fibers, both when sense and antisense probes were employed. In ovaries and eggs, staining was only seen in sections with the antisense probe, detecting viral mRNA. All lice stained positive for at least one of the two viruses (results not shown).

### Cell culture

All the tested cell cultures, BF-2, CHSE-214, ASK and RT-Gill-W1, appeared to be refractory to the two rhabdoviruses from salmon louse.

## Discussion

Rhabdoviruses infect a variety of hosts such as mammals, fish, birds, reptiles, insects, crustaceans and plants [42], [43], [44], [45], [46], [47], [48], [49], [50], [51], [52], [53], [54]. They have evolved different modes of transmission including transmission by arthropods, through direct contact, through gametes and through water. Eleven genera of rhabdoviruses are recognized where viruses associated with arthropods and a wide range of vertebrates, including fish, are found within the genera Vesiculovirus, Ephemerovirus, Sprivivirus, Sigmavirus, Tibrovirus, Tupavirus and some unassigned rhabdoviruses (dimarhabdovirus super group [55]). This is the first study where the nearly complete genomic sequences of new rhabdoviruses obtained from a parasitic copepod, *Lepeophtheirus salmonis*, are presented. Phylogenetic analysis of the two salmon louse viruses, LSRV-No9 and LSRV-No127, based on the L and N protein clearly places them as distinct virus species among these members of *Rhabdoviridae*. The significant divergence of the two lice viruses compared to the closest members of *Rhabdoviridae* suggests that they probably deserve to be recognized as a new genus within this family.

The gene organization, 3′-N-P-M-G-L-5′, is the same as for members of *Vesiculovirus* [47]. There are no additional genes interposed between the five structural genes, as found in some genera of the Rhabdoviridae [56], [57], [58], [59], [60]. The RNA binding motif (G(L/I)SXKSPYSS) sequences that are relatively conserved among N proteins from vesiculoviruses, ephemeroviruses and lyssaviruses [61], [62], [63] are also present in a conserved area in the central region of both louse viruses N protein. The P and M proteins of the two salmon louse viruses show little or no similarity to other described rhabdoviruses, while the G protein of the two salmon louse viruses, like that of other rhabdoviruses is predicted to be a class I transmembrane glycoprotein with an N-terminal signal peptide, glycosylated ectodomain, a transmembrane domain and a short C-terminal cytoplasmic domain [47]. The L protein of the two louse viruses have identifiable sequence homology to other rhabdoviruses, containing all six conserved regions, and associated motifs; RNA template binding, RNA-dependent RNA polymerase, mRNA capping, polyribonucleotidyltransferase activity, methyl transferase activity, and polyadenylation/protein kinase activity [41]. The amino acid sequences of the L protein show close to 40% identity to *Vesiculovirus*. Hence the gene organization and the most conserved genes and motifs support that the two louse viruses belong in the family *Rhabdoviridae*. The non-coding gene junctions of the two salmon louse viruses also contained the conserved transcription termination/polyadenylation motif TATG(A)^7^ and the relatively conserved transcription initiation motif AAGAA/G found among other related rhabdoviruses [43], [45], [46], [49], [50], [51], [53], [57], [60], [64].

Although arthropods are frequently involved as hosts of rhabdoviruses, only a few have been associated with crustaceans and none characterized from parasitic copepods [7], [10], [14], [16], [44], [49], [50], [51], [52], [53], [56], [58]. The salmon louse (*L. salmonis*), parasitizing salmonids in the northern Atlantic and Pacific oceans, is one of several blood feeding fish parasites found among crustacean copepods. Screening of *L. salmonis* collected in Norwegian salmon farms for presence of the two louse viruses, show that all stages including the egg strings of this parasite are positive for both viruses, and *in situ* hybridization and transmission electron microscopy show that the two viruses are present in glandular tissues of adult lice. The ovaries are also positive in the *in situ* hybridization test, but rhabdovirus virions were not observed using TEM on this organ. The host (*S. salar*) for the salmon louse seems to be negative for presence of these two viruses and it has not been possible to culture these viruses in cell cultures obtained from salmonids. The weak positives (Ct values >30) found during screening of skin and gills could possibly be a result of contamination from salmon lice present on the fish. Still, relatively low Ct values were obtained when skin tissues from the Atlantic salmon at the attachment sites for the chalimi stages of the salmon louse were tested. This could suggest that the louse injects the virus into the host skin during the attachment process which would explain the presence of virions in the mandibular glands of the parasite. It is also tempting to speculate that this could be part of a strategy used by the louse to prevent the rejection of the frontal filament that the louse injects into the host skin during early establishment on the host. It has been shown that bites from arthropods can modulate vertebrate host functions by several mechanisms including modulation of the immune response and vasodilation [65]. If this is the case then this group of viruses could be present in most members of the *Caligidae* (a large group of fish parasites). Sequence comparisons, using the N protein from LSRV-No9 and the G protein from LSRV-No127, indicate that similar viruses are most likely also present in parasitic copepods in the Pacific Ocean. The nucleotide sequence from the N protein ORF of LSRV-No9 shows 89.9% identity to a N protein ORF obtained from subspecies *L. salmonis onchorhynci* [66] in Canadian waters, while the G protein ORF from LSRV-No127 shows 50.9% identity two a sequence obtained from *C. rogercresseyi* (Accession no: BT075815) in Chilean salmon culture. Rhabdoviruses and rhabdovirus-like particles have also been detected in glandular tissues of other arthropods and crustaceans [10], [11], however, nothing is known about the genome of viruses from these other crustaceans.

The two rhabdoviruses characterized in this study are the first members of this family that infect copepods, however, there are reports suggesting that spring viraemia of carp virus (SVCV) could be transmitted by a fresh water crustacean, the fish parasite *Argulus foliaceus* [67]. SVCV has also been isolated from crustaceans, *Penaeus stylirostris* and *P. vannamei*, causing mortalities in both fish and penaeid hosts [16]. It has been shown that the salmon louse (*L. salmonis*) may function as a mechanical vector for infectious salmon anaemia virus (ISAV) and infectious haematopoietic necrosis virus (IHNV) [68], [69], [70], and recently, it was shown that another Caligidae, *Caligus rogercresseyi*, may function as a mechanical vector for ISA virus in the culture of Atlantic salmon in Chile [71]. However none of these viruses have been demonstrated to replicate in these parasitic copepods. Rhabdoviruses have been isolated and detected in several fish species including salmonids like *Salmo trutta* and *S. salar* [64], [72], [73], [74], but these viruses are genetically distant from the two salmon louse rhabdoviruses which are not associated with any disease in Atlantic salmon.

## Conclusions

The present study characterize the genome of two new rhabdoviruses obtained from the parasitic copepod *Lepeophtheirus salmonis*, identify their target tissues by *in situ* hybridization, and their putative virion morphology by TEM. Comparison of the genomes show that the two viruses cluster among the Dimarhabdovirus/Sigmavirus groups as two distinct new species that might be classified as distinct from the 11 currently recognized *Rhabdoviridae* genera. The gene organization, 5′-N-P-M-G-L-3′, of the two viruses is the same as that described from *Vesiculovirus*.

Detection of substantial amounts of RNA from both lice viruses at the attachment site for the parasite at the salmonid host suggest that the louse injects the viruses into the skin during early establishment on the host. If the salmon louse uses these viruses for modulation of the immune response in the salmonid hosts one can expect that the other fish parasite species in the copepod family *Caligidae* could be using related viruses for the same purpose. This hypothesis is supported by the presence of a G protein gene, showing high similarity to the G protein from the two salmon louse viruses in the parasitic copepod *Caligus rogercresseyi* collected in the South Pacific Ocean. The existing large diversity of the *Rhabdoviridae* is underscored by the uniqueness of these two viruses from the salmon louse and suggests that more studies are needed to map the complexity of this virus family.
